# Pembrolizumab: An Immunotherapeutic Agent Causing Endocrinopathies

**DOI:** 10.7759/cureus.8836

**Published:** 2020-06-25

**Authors:** Abdul Chaudry, Misbat Chaudry, Jonaid Aslam

**Affiliations:** 1 Cardiology, University of North Carolina, Chapel Hill, USA; 2 Hematology Oncology, Lehigh Valley Health Network, Allentown, USA; 3 Pulmonology and Critical Care, Lehigh Valley Health Network, Allentown, USA

**Keywords:** pembrolizumab, keytruda, immune-related adverse effects, type i diabetes mellitus, autoimmune thyroiditis

## Abstract

Pembrolizumab is an immune checkpoint inhibitor (programmed cell death 1) approved for use in non-small cell lung carcinoma (NSCLC). Pembrolizumab has shown remarkable results in regression of the size of tumors in NSCLC and has shown survival advantage. However, immune-related adverse effects are a serious negative outcome of therapy. The number of immune-related adverse effects with pembrolizumab has increased significantly over the recent past. We present a case of type 1 diabetes mellitus and autoimmune thyroiditis with pembrolizumab treatment for NSCLC.

## Introduction

Pembrolizumab is a monoclonal antibody responsible for inhibiting the ligand of programmed cell death 1 (PD-1) receptor on T-cells. This practice-changing addition to tumor management led to a regression of tumor size in melanoma and non-small cell lung (NSCLC) cancer patients. Despite noteworthy beneficial effects of pembrolizumab in the treatment of the forementioned tumors, it is also associated with immune-related adverse effects (irAEs), including hypophysitis, colitis, thyroiditis, pneumonitis, hepatitis, and type 1 diabetes mellitus (T1DM). To the best of our knowledge, the occurrence of T1DM and thyroiditis simultaneously in a patient after pembrolizumab therapy has not been reported yet. We are reporting a case of a patient who developed thyroiditis and T1DM as an adverse effect of treatment with pembrolizumab.

## Case presentation

A 75-year-old non-diabetic male diagnosed with relapsed NSCLC on follow-up imaging for left lower lobectomy for early-stage lung cancer. Given his excellent performance status, he was deemed a candidate for chemotherapy plus immunotherapy and started on carboplatin, pemetrexed, and pembrolizumab. The interim scan showed a response to treatment. After three cycles of chemotherapy, he developed fatigue, severe nausea, and weight loss. His labs revealed neutropenia with absolute neutrophil count (ANC) of 300/mm^3^ after the third cycle. Carboplatin was held as a result, but pemetrexed and pembrolizumab were continued for a fourth cycle. Fatigue persisted, and he developed dyspnea on exertion. He was taken to the emergency department and found to be in atrial flutter with a heart rate of 154 bpm. A comprehensive metabolic panel revealed the patient to have a blood glucose of 642 mg/dl, with a serum bicarbonate level of 12 mEq/L and an anion gap of 20. Lactate was normal at 1.9 mg/dL, and creatinine mildly elevated at 1.37 mg/dL. A diagnosis of diabetic ketoacidosis was made, and he was started on intravenous fluids and an insulin drip. Further workup revealed increased levels of antibodies to glutamic acid dehydrogenase (anti-GAD), thyroid peroxidase (anti-TPO), and thyroglobulin autoantibodies (563 IU/mL). These findings were consistent with the diagnosis of T1DM and autoimmune thyroiditis and presumed to be an adverse effect of pembrolizumab therapy.

Intervention

Treatment with pembrolizumab and pemetrexed was held because of the occurrence of these serious adverse effects. He is on a subcutaneous insulin regimen to manage his T1DM. A three-week follow-up is planned, and if asymptomatic, pemetrexed only will be continued, with pembrolizumab being discontinued due to adverse events.

## Discussion

Pembrolizumab is a monoclonal antibody responsible for inhibiting the ligand of PD-1 receptor on T-cells [1]. It was approved by Food and Drug Administration (FDA) in 2015 for metastatic NSCLC [2]. Despite the impressive effects of pembrolizumab on improving survival in NSCLC, its use can be restricted by severe irAEs. The most common adverse events include pneumonitis, colitis, hepatitis, hypophysitis, thyroiditis, and nephritis [3]. T1DM is a relatively rare adverse effect and only occurs in 0.1% of patients.

The use of immune checkpoint inhibitors (ICIs) like pembrolizumab is related to severe irAE. T1DM and autoimmune thyroiditis are rare side effects of pembrolizumab, and the occurrence of both these adverse effects at the same time is even more unique.

Diagnosing T1DM in patients taking pembrolizumab can sometimes be challenging because elevated levels of anti-GAD antibodies are present in only half the cases. Although the presence of anti-GAD antibodies is not necessary for the diagnosis of T1DM, it does confirm the diagnosis [4].

The management of irAEs includes discontinuation of pembrolizumab and using glucocorticoids. The first-line management of irAEs is 1-2 mg/kg/day methylprednisolone IV for two to three days and then conversion to oral prednisolone with weaning off in four to six weeks to minimize immune-related destruction of organ systems. The problem of using steroids in T1DM is that it does not have any impact on the outcome of disease, and increases insulin resistance and promotes hyperglycemia [5]. Moreover, T1DM, in this case, was easily managed by insulin therapy.

## Conclusions

ICIs, i.e., pembrolizumab, are helpful in regression of tumor size in NSCLC but irAEs, i.e., T1DM and autoimmune thyroiditis, are serious events to watch out for. If immunotherapy is considered in patients of NSCLC, continuous monitoring of blood glucose levels and thyroid-stimulating hormone (TSH) is necessary as these adverse events can prove life threatening and result in fatalities.
